# Depression alters the circadian pattern of online activity

**DOI:** 10.1038/s41598-020-74314-3

**Published:** 2020-10-14

**Authors:** Marijn ten Thij, Krishna Bathina, Lauren A. Rutter, Lorenzo Lorenzo-Luaces, Ingrid A. van de Leemput, Marten Scheffer, Johan Bollen

**Affiliations:** 1 Luddy School of Informatics, Computing and Engineering, Center for Social and Biomedical Complexity, Indiana University Bloomington, Bloomington, IN 47408 USA; 2 Department of Psychological and Brain Sciences, Indiana University Bloomington, Bloomington, IN 47405 USA; 3 Aquatic Ecology and Water Quality Management, Wageningen University, Wageningen, 6708 PB The Netherlands

**Keywords:** Psychology and behaviour, Computational science, Computer science

## Abstract

Human sleep/wake cycles follow a stable circadian rhythm associated with hormonal, emotional, and cognitive changes. Changes of this cycle are implicated in many mental health concerns. In fact, the bidirectional relation between major depressive disorder and sleep has been well-documented. Despite a clear link between sleep disturbances and subsequent disturbances in mood, it is difficult to determine from self-reported data which specific changes of the sleep/wake cycle play the most important role in this association. Here we observe marked changes of activity cycles in millions of twitter posts of 688 subjects who explicitly stated in unequivocal terms that they had received a (clinical) diagnosis of depression as compared to the activity cycles of a large control group (n = 8791). Rather than a phase-shift, as reported in other work, we find significant changes of activity levels in the evening and before dawn. Compared to the control group, depressed subjects were significantly more active from 7 PM to midnight and less active from 3 to 6 AM. Content analysis of tweets revealed a steady rise in rumination and emotional content from midnight to dawn among depressed individuals. These results suggest that diagnosis and treatment of depression may focus on modifying the timing of activity, reducing rumination, and decreasing social media use at specific hours of the day.

## Introduction

Depression is one of the most important global public health challenges. It is the single largest contributor to disability and disease, affecting 4% of the world’s population, causing 11% of all years lived with disability globally^1^. It is furthermore associated with a reported 800,000 suicides on an annual basis, mostly among young adults^2^. Depression is significantly under-reported, under-diagnosed, and under-treated, in part due to its heterogeneous nature which involves subjective and culturally shaped experiences such as motivation, mood, and well-being^3^. Furthermore, in spite of its prevalence, the dynamics of its onset and development remain poorly understood^4–6^, limiting the development of treatment options^7,8^.

Like most mammals^9^, humans experience circadian rhythms involving hormonal, behavioral, and cognitive changes that lead to stable sleep-wake cycles, even when individuals are disconnected from natural daylight^10,11^ or travel across time zones. Unsurprisingly, a stable daily activity cycle is important to maintain physical and mental health^12,13^. In fact, disturbances of the human circadian rhythm are strongly associated with mood disorders^14–22^ such as depression and anxiety, bipolar, and borderline personality disorder. The severity of depression has been linked to the magnitude of the sleep-wake cycle disturbance^23^ while reports of sleep disturbances can be used as an early warning signal of recurrent depression^24^ and predict risk of poor outcomes in treatments for depression^25^. As a result, interventions targeting sleep are now considered an essential component of efforts to improve depression treatment outcomes^26,27^. This is also emphasized by the central position of sleep-related symptoms in disorder networks^28^.

Although the connection between sleep-wake cycle disturbances and depression has been firmly established, it is not clear which specific disturbances or changes are most strongly implicated in the onset and remission of depression. Reports of the effectiveness of sleep deprivation therapy^29,30^ indicate that the association between sleep and mood disorders is not necessarily modulated by the amount of sleep per se^31^, but by its specific timing and pattern. In particular, questions have arisen with respect to whether *phase* and/or *magnitude* changes of the sleep-wake cycle account for the association between sleep and risk for depression^32^.

Observations of daily activity levels of individuals require continuous monitoring of a large number of subjects throughout numerous circadian cycles to establish sufficient statistical power while avoiding observer bias. However, most studies establishing circadian rhythm disturbances in mental disorders suffer from small sample sizes^33^. These limitations can be mitigated by the *post hoc* analysis of alternative sources of information such as microblogs, diaries, mobile phone^34^, and social media activity. The latter in particular serve as a daily cognitive and behavioral diary to billions of individuals. In fact, activity levels in on-line platforms, e.g. using Digg^35^, Foursquare^36^, Twitter^37^, Wikipedia editing behavior^38^, and YouTube^35^, have already proven to be a useful resource to estimate circadian cycles.

Here, we use large-scale, longitudinal, social media activity data to study the daily activity cycles of hundreds of individuals who stated in unequivocal terms that they had received a (clinical) diagnosis of depression, using an similar sample inclusion criterion as Coppersmith, Dredze & Harman^39^. We find that the activity levels of depressed individuals, like those of a random sample, fluctuate reliably according to a well-defined circadian rhythm as was shown previously^40^. Our results extend these findings by showing no evidence of a significant phase-shift, but rather that activity levels for the depressed individuals differ significantly in the early evening and early morning hours, which is when we also see increased indications of emotionality and self-reflection. These findings point towards targeted interventions that focus on the reduction of rumination at specific times of the day.

## Cohort definition

For our analysis, we define 2 disjoint cohorts of Twitter users: “Depressed” and “Random”. In our “Depressed” cohort we only include individuals with a (clinical) diagnosis of depression, which they report on Twitter explicitly (e.g., “Went to my doctor today and got officially diagnosed with major depression”), similar to the approach of Coppersmith, Dredze & Harman^39^. A team of 3 raters independently evaluated each ‘diagnosis tweet’ to determine whether it pertained to an explicit, unequivocal statement of an actual diagnosis, removing self-diagnoses, retweets, quotes, or jokes. In other words, we excluded individuals who “self-diagnosed” with depression. This second step was taken to remove false-positives from the cohort, which has been proven to increase performance in classification tasks^41^. We also mapped references to a time of diagnosis, e.g. “today”, “last week”, “2 months ago”, or “in 2014” to a likely diagnosis time interval (see “Methods”). This method is akin to research on electronic health records (EHRs) as well as pharmacoepidemiological methods in the sense that we rely on reports of an actual diagnosis but are receiving this information directly from the individual with the diagnosis. This allows us to tie the diagnosis to their social media record, which provides indicators of their evolving mood, cognition, language, and behavior. While the recognition of depression is poor in some settings^42^, patients who are recognized as being depressed tend to, on average, have higher levels of depression than those who are not recognized^43^. This finding, along with research suggesting depression is best understood as existing on a continuum (for a review see Ruscio^44^), supports the validity of our inclusion criteria for the “Depressed” cohort. We found 688 individuals that explicitly stated their (clinical) depression diagnosis and whom we assigned to the “Depressed” cohort, or *D* cohort for short. We downloaded the past tweets of these aforementioned individuals to obtain a longitudinal timeline.

Neither the reported diagnosis nor the Twitter profiles of the sampled individuals provide demographic information with respect to our *D* cohort. However, a highly accurate sex classifier^45^ (Macro-F1: 0.915) applied to the Twitter profiles of our *D* cohort (see “Methods”), shows that it has a similar 2:1 female to male ratio as observed in clinical studies^46^, indicating that the demographics of our Twitter cohort closely match previous clinical findings. The indicated age distribution of our *D* cohort (though less reliable, Macro-F1: 0.425), is also in line with clinical studies^46,47^, specifically we find a decreasing number of individuals per age-group as the age of the group increases in our *D* cohort.

**Table 1:** Demographic information derived with M3^45^ for both cohorts.

All individuals		“Depressed” cohort	“Random” cohort
		688	8791
Gender	Male	181	3313
	Female	356	2,918
Age	18 and under	31	613
	19–29	78	659
	30–39	58	538
	40 and over	41	1,006

We define our “Random” cohort, or *RS* cohort for short, as a control group by taking a random sample of 8791 Twitter users. To compensate for possible changes of user behavior in the social media platform over time, we sample these individuals such that the distribution of their account creation month matches that of the individuals in the *D* cohort (see Supplementary Information Section 2). Table 1 describes the demographic information obtained for both cohorts.

## Measuring activity levels

We assume that sleeping individuals can not tweet and that we can therefore gauge changes in activity levels by counting the number of tweets that an individual posts at a given time. Working at an hourly resolution, we count the number of tweets that an individual has posted at a given hour of the day and divide each hourly count by the total number of tweets for all hours of the day. This results in an hourly percentage of daily Twitter activity for the individual (denoted ${\mathscr {A}}_u$). We can then calculate a cohort hourly activity level for either the *D* cohort or the *RS* cohort, denoted ${\mathscr {A}}_D$ or ${\mathscr {A}}_{RS}$, respectively, by combining all hourly counts across the individuals in the specific cohort and dividing by the total number of tweets across these individuals. Note that we exclude retweets and account for each individual’s local time to ensure counts pertain to the same time of day.

Naturally, differences can arise in the level of activity between both individuals and cohorts in general. Since we are not looking to make inferences about the total amount of tweets nor the average number of tweets per cohort, but rather the relative differences of hourly activity patterns between the two cohorts, we account for this variation by calculating hourly activity levels for 10,000 re-samples of the individuals in the *D* and *RS* cohorts with replacement, i.e. we bootstrap hourly activity levels for each cohort. This re-sampling results in a distribution of activity levels for each hour (each from a different sample of individuals) that can be characterized by its median and 95% confidence interval, denoted by ${\mathscr {A}}^\star _D$ and ${\mathscr {A}}^\star _{RS}$ respectively for the *D* and *RS* cohort.

**Figure 1:**
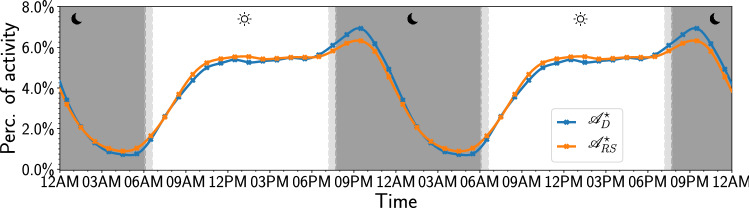
Bootstrapped normalized activity levels for the “Depressed” and “Random” cohorts. The markers display the median outcome of 10,000 runs, where we use the number of individuals in each cohort as the sample size per run ($n=688$ for the “Depressed” and $n=8791$ for the “Random” cohort). The solid lines display the cubic spline fit of these hourly values. The dark and light gray shaded areas indicate the day/night times during the cycle (see “Methods”).

**Figure 2:**
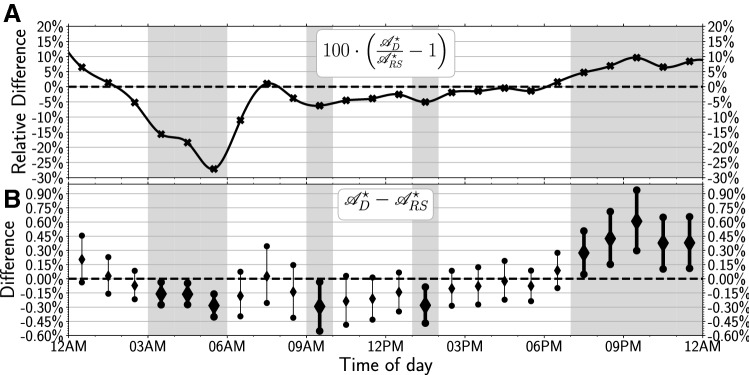
Bootstrapped difference between the normalized activity levels for the “Depressed” and “Random” cohorts. (**A**) Relative difference between the “Depressed” and “Random” cohorts. The markers indicate the hourly relative difference between the mean activity levels (see Fig. 1) for both cohorts and the solid black line displays the cubic spline fit of these hourly values. (**B**) Bootstrapped difference between the “Depressed” and “Random” cohorts. The diamonds display the median outcome of the difference in outcome of the 10,000 runs and the vertical lines display the 95% CI of the difference in the bootstrap outcomes. The hours displayed in bold indicate that there is a significant difference in behavior between the two cohorts. Furthermore, the gray shaded areas in both panels indicate the hours in which there is a significant difference in activity and the black dashed lines in both panels are meant as a reference lines that indicate equal behavior for both cohorts.

**Figure 3:**
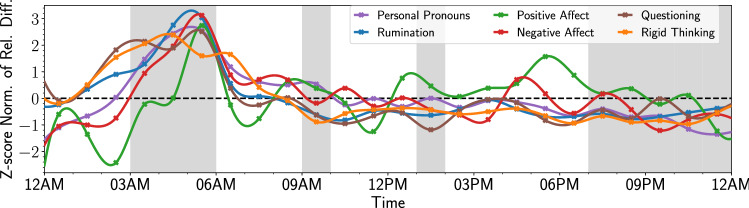
Z-score normalized relative difference in token usage between the “Depressed” and “Random” cohorts. The Z-score normalized hourly values of $PR^h\left( {{\mathscr {C}}_x}\right)$ for all selected tokens and each category are indicated by the colored markers (see SI Section 5 for the actual values). The solid lines display the cubic spline fit of the hourly values. The black dashed line is a visual representation of the mean behavior. Furthermore, the gray shaded areas indicate the hours in which there is a significant difference in activity.

## Circadian activity levels

The resulting time series ${\mathscr {A}}^{\star }_D$ and ${\mathscr {A}}^{\star }_{RS}$ are displayed in Fig. 1. As a reference to aid the eye, we show the times of dawn, sunrise, sunset and dusk as gray bands. We repeat the cycle twice in Fig. 1 to better highlight the daily variation around midnight.

For both the *D* and *RS* cohorts, we find periodic changes in activity levels throughout the day, resulting in a well-defined circadian rhythm of activity levels. We find that both cohorts experience a valley in activity levels from roughly 10PM to 6AM, a time that is traditionally reserved for sleep. Activity levels quickly recover from a low point at 6AM as people wake up and become active during the morning hours. This is followed by a first peak at noon, after which activity plateaus for 6 h from noon to 6 PM. This is followed by a slight ramp up of activity peak around 9 PM, after which activity levels drop again.

Tweets can be posted at any time of year, hence seasonal changes in daylight times or Daylight Savings Time could affect our observations. However, we find that daylight times changes throughout the year do not account for our pattern of results (see Section 3.2 of the Supplementary Information).

## Differences in activity levels between “Depressed” and “Random” cohorts

As shown in Fig. 1, activity levels of the *D* and *RS* cohorts follow a similar circadian rhythm with valleys and peaks occurring at approximately the same time. We find no evidence of a phase-shift in daily activity levels; the pattern of changes, including the valleys and peaks of the circadian rhythm, match exactly across the *D* and *RS* time series. A cross-correlation function indicates that the Pearson correlation coefficient between the two time series peaks exactly at a lag of zero (see Supplemental Information Section 4), providing further indication of the absence of a phase-shift between the sleep/wake cycles of the *D* and *RS* cohorts.

However, in spite of the absence of a phase shift in Fig. 1, we do find that activity levels diverge significantly at specific times of day between the *D* and *RS* cohorts. In particular, we find divergences from 3AM to 6AM, 9AM to noon, and a particularly sharp divergence from 9PM to midnight. In the latter case, surprisingly, we observe that the *D* cohort is approximately 1% *more active* than the *RS* cohort, a considerable amount relative to the expected range of percentage-wise hourly fluctuations throughout the day for both cohorts, namely roughly 1% to 8% from peak to valley and an expected 4.16% hourly activity if uniformly distributed over 24 h ($100\% / 24 \simeq 4.16\%$).

To objectively determine the significance of the observed differences between the circadian activity levels of the *D* and *RS* cohorts, we calculate the hourly relative differences of activity levels between the two cohorts, i.e. the ratio of activity levels at hour *i* between the *D* and *RS* cohort. If this ratio equals 1 we assume the activity levels are equal. Activity level ratios significantly larger or lower than 1 indicate a significant difference in activity levels.

Figure 2A shows that this relative difference is lowest at 5AM and highest at 9 PM, i.e. individuals in the *D* cohort are much less active in the early morning ($-27\%$ from 3 to 6 AM) but more active in the evening ($+10\%$ from 7 PM to midnight) compared to individuals from the *RS* cohort.

Our cohorts are comprised of individuals with different activity levels. It follows that the inclusion or exclusion of individuals in both cohorts will affect our estimate of activity level differences. This should be taken into account when we assess whether or not activity levels are significantly different at a particular hour between the two cohorts. We therefore bootstrap the difference between the two activity levels (${\mathscr {A}}_D - {\mathscr {A}}_{RS}$), by re-sampling the individuals in both cohorts with replacement. This results in a distribution of difference values that we can characterize by its median and 95% confidence interval (CI), as shown in Fig. 2B. If the resulting 95% CI does not include 0, we conclude that the activity levels for that hour differ between the *D* and *RS* cohorts at the $\alpha <0.05$ level^48^.

According to this criterion, we find the following statistically significant divergence of circadian activity levels: *less* activity for the *D* cohort between 3 and 6AM, 9 and 10AM, and 1 and 2PM, as well as *more* activity in the evening between 7PM and midnight for the *D* cohort. These times of significant differences are also marked in Figs. 2 and 3 by the gray shaded areas. Strikingly, the differences in the morning hours do not coincide with the distribution of dawn or sunrise times, which we determined for the location of all individuals in each cohort using their self-reported location information (see “Methods”). This indicates that the differences are not caused by variances in the response to daylight hours.

## Content analysis

The circadian activity levels of the *D* and *RS* cohorts differ significantly at specific times of the day. To investigate the cognitive factors that may affect these differences, we analyze the content of the tweets posted by the individuals in both cohorts at those times when activity levels diverge significantly.

Two experts in cognitive-behavioral therapy (CBT) selected a set of 76 tokens (listed in Table 2), each falling into six different categories (denoted by ${\mathscr {C}}_x$) related to “self-reflection” and “rumination” (e.g. Personal Pronouns, where $x=PP$ so ${\mathscr {C}}_{PP}$), with a set of tokens expressing “positive affect” as a control. We define the prevalence of a token *t* in a cohort (i.e., *D* for “Depressed” or *RS* for “Random”) as the expected number of times that the token is used per tweet (denoted by $f_D\left( t\right)$ and $f_{RS}\left( t\right)$, respectively). The first column of Table 3 shows that the token prevalence ratio between the *D* and *RS* cohorts for all considered categories (denoted by $PR({\mathscr {C}}_x)$) is much larger than 1.

**Table 2:**
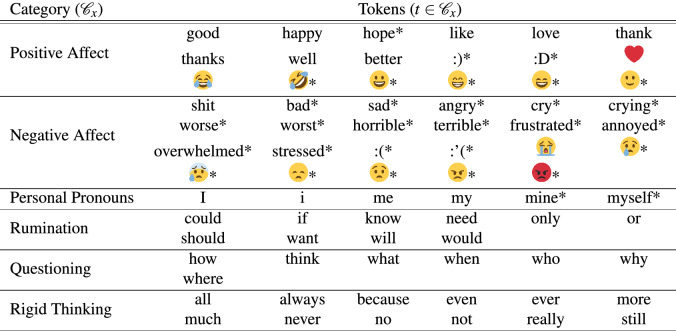
Overview of tokens used for content analysis.

An * indicates that a token was not consistently in the top 250 tokens for each hour in both cohorts.

**Table 3:** Prevalence ratios in token use for all considered categories.

Category (${\mathscr {C}}_x$)	Prevalence ratio ($PR\left( {\mathscr {C}}_x\right)$)	Hourly prevalence ratio ($\frac{1}{24}\sum _{h=0}^{23}PR^h\left( {{\mathscr {C}}_x}\right)$)
All selected tokens	1.4885	1.5279
Personal pronouns	1.7601	1.8217
Positive affect	1.4593	1.4589
Negative affect	1.6982	1.7666
Rumination	1.2455	1.2836
Questioning	1.2319	1.2618
Rigid thinking	1.3467	1.3922

Like our activity level time series, we analyze token prevalence ratio on an hourly basis for each category of tokens separately. Figure 3 displays the z-score normalized time series of hourly token prevalence values. These time series show significant changes in token use for the *D* cohort from 3AM to 6AM, which occurs in conjunction with *lower* activity levels for the *D* cohort, as indicated by the shaded areas.

More specifically, we see a drop in positive affect and an increase in Rigid Thinking and Questioning from midnight to 3AM. This is followed by an increase in the use of tokens associated with Personal Pronouns and Negative Affect from 4AM to 6AM. Token use across all categories peaks from 5AM to 6AM, the early morning hours, which indicates higher levels of “rumination” and “self-reflection” among individuals in the *D* cohort. Since our tokens were designed to indicate “self-reflection” and “rumination” (with the exception of Positive Affect), this pattern is indicative that wakefulness at that time is associated with negative psychological states. The increase in usage of both Positive and Negative Affect may be indicative of higher levels of emotionality.

The time series in Fig. 3 are z-score normalized (centered around 0, dashed line in Fig. 3) to highlight changes in prevalence over time. Since these token categories focus on language that contains “rumination” and “self-reflection”, all categories are more prevalent in the *D* cohort than the *RS* cohort. In addition, mean prevalence values per token category can vary considerably, i.e. some categories of tokens occur more frequently than others *throughout the day* and hourly as shown in Table 3.

## Discussion

Comparing hourly Twitter activity levels for two cohorts of respectively “Depressed” and “Random” individuals, we find significant differences in the activity patterns of depressed Twitter users vs. a random sample. Unlike previous studies, we observe no phase-shift between the circadian rhythms of the *D* and *RS* cohorts, but rather significant differences in the magnitude of activity levels at specific times. As shown in Fig. 2, the *D* cohort is significantly less active from 3 AM to 6 AM and significantly more active from 7 PM to midnight than the *RS* cohort.

The latter difference corresponds to an interesting daily peak of highest activity at 9 PM which occurs in both cohorts. This peak of activity levels may correspond to a period of time after dinner and before bedtime which individuals use for recreational social media use^49^. Since this peak is more pronounced for the *D* cohort, it raises the interesting possibility that social media use is partly involved in altering the circadian activity levels of the *D* cohort. As such, in this case, we are not merely observing a change in circadian rhythms, but the differential effects of social media.

Comparing tweet content at specific times between the *D* and *RS* cohort shows that the *D* cohort more frequently use tokens that relate to “self-reflection” and “rumination” than the *RS* cohort. Furthermore, Fig. 3 shows that individuals in the *D* cohort who are awake between 3 AM and 6 AM show higher levels of “rumination” and “self-reflection”. Moreover, the simultaneous rise in both Positive and Negative Affect tokens at this time may be indicative of a higher level of emotionality in the *D* cohort. Since the *D* cohort is less active at a given time, we note that our content analysis only captures the activity of individuals that are still awake.

The fact that we find more frequent usage of the majority of tokens across all six categories suggests that depressed individuals, when awake, post messages that contain higher levels of so-called “depressogenic” language. Therefore, by analyzing the corresponding patterns around the chosen tokens we could gain insight into differences in broader language use between depressed individuals and the general population. These difference could be leveraged in the development of treatments as well as early intervention, in particular in the context of cognitive-behavioral therapy (CBT) which involves a significant cognitive and linguistic component. Our current analysis first and foremost aims to establish the difference in activity levels and associated language use between the described cohorts, hence we leave this analysis for future work.

We caution that although we verified that the individuals in our *D* cohort explicitly stated that they received a clinical diagnosis of depression, we have no ground-truth verification of the veracity of these statements, nor do we have specific information about the time of the diagnosis for all individuals in the *D* cohort. Based on the time indications in some the expressions, e.g.  “yesterday” or “last week”, we were able to determine an interval in which the diagnosis occurred for only 93 individuals in the *D* cohort (out of 688). However, $83.87\%$ of these 93 individuals stated that the diagnosis occurred within a year of the diagnosis tweet indicating that the episode of depression was relatively recent (and within diagnostic boundaries) for many individuals in this cohort.

It is nevertheless inevitable that our *D* cohort will contain a mix of individuals with recent or past diagnoses, cases that have either been resolved or remain unresolved, or co-morbid disorders. Likewise, our *RS* cohort may contain depressed individuals who did not explicitly state a diagnosis, but suffered from depression in the past or the present. Even though our *D* cohort is likely to contain a significantly greater number of individuals that suffer from depression than our *RS* cohort, both cohorts may be heterogeneous to some degree. Such heterogeneity would decrease the observed differences between the groups and increase error, thus tending to reduce statistical power, not increase it. As a result, it would not call into question the validity of our results. We furthermore carefully bootstrapped all activity estimators to estimate the sensitivity of our results to sample heterogeneity.

Our findings illustrate how social media data can be leveraged to investigate the longitudinal and individual effects of mental disorders on circadian sleep/wake cycles, complementing insights obtained through traditional methods. Based on our results, social media data offers distinct opportunities for this field of research. First, it allows for the construction of large cohorts to be analyzed with higher statistical power with respect to changes in circadian rhythms, and second, the fact that the tweets are analyzed ex post hoc ensures that the results are not influenced by the Hawthorne effect^50^. The value of social media data can be augmented further with other sources of mental health information, such as electronic health records, which can be cross-validated to social media text with the appropriate use of machine learning and AI algorithms. Third, studies of the online patterns of activity of depressed individuals may inspire new opportunities for intervention, for example with CBT for insomnia, delivered efficiently over the internet^51^. Finally, social media, regardless of its utility as data sources for social science^52–55^, has become an important factor in the social lives of billions of individuals. The analysis of social media and related mobile communication data might therefore shed light on how or whether these platforms affect public health at a global scale^34,49,56^.

## Methods

### Data and sample

In our “Depressed” cohort we only include individuals with a (clinical) diagnosis of depression, which they report on Twitter explicitly (e.g., “Went to my doctor today and got officially diagnosed with major depression”). To obtain the widest possible set of tweets that could contain such a statement, we searched the Twitter search Application Programming Interface (API) and the IUNI Observatory on Social Media (OSoMe)^57^ for tweets that were posted between Jan 1st 2013 and Jan 1st 2019, and contained the terms: diagnos* and depress* (* indicates “any following characters”). We found 4,002 such tweets posted by 3,324 unique Twitter users. Three of the co-authors manually and independently rated the content of each such tweet in terms of whether it did in fact contain a valid, unequivocal, and explicit statement of a (clinical) depression diagnosis, removing matches resulting from jokes, quotes, self-diagnoses, or non-self-referential statements, e.g. “a friend just told me...”. In other words, we excluded individuals who “self-diagnosed” with depression. We thus obtained a list of 1,211 individuals that explicitly expressed that they received a (clinical) diagnosis of depression.

Next, for each individual who posted a ‘diagnosis’ tweet, we harvest their public profile information and a timeline of their past tweets (up to the allowable maximum of the 3,200 most recent tweets at the time of collection) from Twitter’s API. The post dates and times of these tweets range from 2008-10-22 01:23:03+0000 to 2018-09-12 12:48:04+00:00. For every individual tweet, we retrieve a unique identifier, the content of the tweet, and the coordinated universal time (UTC) at which it was posted. In total, we find 2,928,720 tweets across the timelines of these 1,211 individuals.

Since we want to compare the timing of circadian activity levels between groups of individuals, we have to localize their respective time zones. We use the self-reported location of the Twitter user’s profile page to determine the time zone of that specific user. Using this approach, we are able to determine a time zone for 690 individuals, for which we obtained a total of 1,686,182 tweets.

Subsequently, we remove tweets that are not posted in English according to the tweet’s language label supplied by Twitter, as well as tweets that match our initial “diagnostic” search criteria so our analysis is not biased by linguistic variables and our sample selection criterion. We also remove all retweets since they are by definition not written by the same individual. We thus retain 1,042,838 tweets that are posted by 688 Twitter users. We will refer to these individuals as the “Depressed” cohort.

### Determining the interval of the diagnosis

For each of the tweets that express a diagnosis, we determine whether their content provides an indication of the Time of Diagnosis (TOD), e.g.  “I was diagnosed with depression *2 weeks ago*”. Not all TODs refer to an exact date on which the diagnosis took place, but rather an interval such as “a couple of weeks ago”. Therefore, we translate these statements to intervals in which the diagnosis most likely happened based on the magnitude and units of time stated by the individual. For example, if an individual stated “2 weeks ago”, this was interpreted as an interval that spans the entire week that took place two weeks before the date of the statement.

Furthermore, if an individual posted several diagnosis tweets with a TOD indication, we use the intersection of all determined intervals, since the diagnosis is most likely to have occurred during this time. If this intersection does not exists, we do not quantify a TOD for this user. In addition, when a tweet text states “I have been recently diagnosed with depression”, we assume that the diagnosis was given in the last three months. We refer the reader to Section 1 of the Supplementary Information for the full list of conversions that we used.

### Constructing a random sample

After we obtained the “Depressed” cohort, we consecutively build a second cohort that consists of random Twitter users as a comparison to the “Depressed” cohort, which we will refer to as the “Random” cohort. Since habits and behavior in platform usage may have changed over time, we construct our random sample in such a way that the distribution of the account creation dates for these individuals (when they started using Twitter) match those of the previously established “Depressed” cohort.

First, we select random tweets from OSoMe. The individuals who posted these tweets were taken as the seed set for the random sample. From this seed set, we then select all Twitter users that have specified their location and that are not in our “Depressed” cohort. We then sample these remaining individuals based on their creation date according to the creation date distribution of the “Depressed” cohort. After these steps, we collect the timelines of the 9,121 individuals we select and obtain 24,992,122 tweets for our random sample data set. These tweets’ post dates range from 2007-08-24 20:36:05+00:00 to 2019-02-08 12:51:43+00:00.

The vast majority of tweets from both cohorts are posted in an overlapping time period of nearly a full decade (2008 to 2018), however there exists a slight variation in the dates of the earliest and latest tweets between the two cohorts. This variation pertains to a minority of tweets and does not affect our results since we are comparing 24 h circadian pattern. The difference in timing of the first observed tweets between the two cohorts is a consequence of the fact that the Twitter API returns the most recent 3,200 tweets for each individual. Since individual activity levels can vary over time, this may lead to slight variations in how far back in time the earliest tweets of a given cohort can be retrieved. The difference in dates of the last tweets recorded for the “Depressed” and “Random” cohorts therefore results from the latter data being retrieved after the former.

After applying the same filtering steps as we used to construct the “Depressed” cohort, our “Random” cohort consists of 8,498,574 tweets that are posted by 8791 Twitter users.

### Demographics analysis

To further quantify our obtained cohorts, we derive the demographic information of all obtained individuals using the M3 system^45^, a deep learning system for demographic inference (gender, age, and individual/person) that was trained on massive Twitter data set using profile images, screen names, names, and biographies. For both the gender- (male and female) and age-dimensions (18 and below, 19-29, 30-39, and 40 and up), we assign an individual we found to a group if the corresponding Twitter account scores above 0.8 on the given label and on the ‘non-org’-label.

### Determining daylight times

To control for the effects of daylight cycles for each of the individuals in our cohorts, we retrieve the user-defined locations of each tweet to derive the time of dawn, sunrise, sunset, and dusk at the location that it was posted from. The inclusion of these times may indicate whether the observed differences in circadian rhythms can arise from differences in the respective daylight cycles that individuals in the “Depressed” and “Random” cohorts experience at their location. Figure 1 displays the median of these times for the “Depressed” cohort. The actual distribution of these times is included in Section 3 of the Supplementary Information, as well as the distribution of daylight times for the “Random” cohort.

### Circadian rhythms construction

For each individual in our cohorts, we determine the circadian rhythm as follows. We localize all creation dates of that individual’s tweets using their respective time zone which was determined from the user-defined location in their Twitter profile. We use these localized post times to determine in which hour of the individual’s local time the tweet was posted. Using this information, we can then produce circadian rhythms for the individuals by binning their tweets based on the localized hour in which they were posted. We normalize the counts of tweets for any given hour to a percentage by dividing the hourly count by the total number of tweets over all 24 h in the day. Hence, for uniform activity levels throughout the day, each hour would have 100% / 24 $\simeq 4.16$% of activity.

### Testing for a phase-shift

In the comparison of the activity levels of the “Depressed” and “Random” cohorts, we also check whether these time series display a phase-shift. We do this by calculating the cross-correlation of the two circadian time series shifted − 12 to + 12 h (wrapped window). The maximum correlation coefficient between the two time series is 0.9929 at a lag of 0 h, which implies the absence of a phase-shift between the time series of our two cohorts (see Section 4 of the Supplementary Information for correlation scores).

### Circadian rhythms bootstrap

We bootstrap the circadian rhythms to determine the uncertainty in our estimation of activity levels that may result from variations in individual activity levels.

This bootstrap consists of 10,000 replications of our calculation of hourly activity levels per cohort. Each replication consists of sampling the cohort with replacement, creating 10,000 re-samples of *n* individuals, and constructing a circadian rhythm for that specific set of individuals combined. Specifically, we sum all counts of hourly tweets for the individuals in the re-sample and then normalize this total by their total tweets, as opposed to normalizing with individual totals as discussed above. Note that each user contributes their hourly tweets to the total for the re-sample, hence the contribution of individuals with low numbers of tweets is minimized in each replication.

To compare the activity levels of two cohorts, we simply calculate the difference between the hourly activity levels obtained in the specific bootstrap replication, leading to 10,000 time series of differences between the circadian rhythms of the two cohorts.

As described above we obtain two bootstrapped circadian rhythms: the hourly activity levels for each cohort separately and the differences between the hourly activity levels between the two cohorts. The hourly distributions can be characterized by their median and 95% CI.

### Tweet content analysis

The tweet texts in our data set are processed as follows. First, the tweets are tokenized using the built-in TweetTokenizer of the *NLTK*^58^ package. Next, all tokens that start with a capital letter and have no further capitalized characters are converted to a non-capitalized form with the exception of “I”. Finally, any contractions that have a unique meaning, e.g. “I’m” or “you’ll” are converted to their non-contracted counterparts. Ambiguous contractions such as “he’s”, which can mean both “he is” and “he has”, are not converted.

### Selecting tokens for the content analysis

To focus our analysis on tokens that are consistently used throughout the day (allowing for comparisons across different times), we determine the 250 most used tokens for each separate hour. Of these tokens, 187 tokens occur in the top 250 tokens for *every hour* of the day. Two of the authors, who are clinical experts in cognitive-behavioral therapy, defined six topic categories as sub-sets of these 187 tokens (See Table 2 which were deemed to be most indicative of the cognitive factors affecting depression, namely (1) “Personal Pronouns”, (2) “Rumination”, (3) “Negative Affect”, (4) “Questioning”, and (5) “Rigid Thinking” with (6) “Positive Affect” as a control. The chosen categories align with feature sets that are found in previous work that analyze the social media content of depressed individuals^40,59^. Next, some tokens were added to complement and balance some of the categories, which are indicated by an asterisk (*) in Table 2. Finally, given their importance in online communication, we added emojis to the positive and negative affect categories, from the classification of https://hotemoji.com/emoji-meanings.html, but only including emoji’s with obvious positive and negative valence, which are also indicated by an asterisk (*) in Table 2. This procedure resulted in 76 tokens distributed over 6 categories.

## Supplementary information

Supplementary Information.

## Data Availability

The datasets generated during and/or analysed during the current study are available in the GitHub repository, https://www.github.com/mctenthij/circadian_rhythms/, allowing reproduction of the results. Additional data and information are available from the authors upon reasonable request.
