# Supplementary material for: Depression alters the circadian pattern of online activity
Source: Sci Rep. 2020 Oct 14;10:17272. doi: 10.1038/s41598-020-74314-3 (PMC7560656; doi:10.1038/s41598-020-74314-3)
Supplement: Supplementary file 1 — Supplementary Information. [file 41598_2020_74314_MOESM1_ESM.pdf]

Supplementary Information: Depression alters the circadian pattern of online activity

Marijn ten Thij[1], Krishna C. Bathina[1], Lauren Rutter[2], Lorenzo Lorenzo-Luaces[2], Ingrid A. van de Leemput[3], Marten Scheffer[3], and Johan Bollen[1,3]

[1]Center for Social and Biomedical Complexity, Indiana University
[2]Department of Psychological and Brain Sciences, Indiana University
[3]Wageningen University, the Netherlands

September 24, 2020

This is the supplemental material for the manuscript titled 'Depression alters the circadian pattern of online activity'. In the course of the manuscript, we touch on various subjects that are not discussed in detail. These subjects are further discussed in this document. First, we expand upon how we determined the intervals in which the diagnoses occurred. Next, we describe the procedure that was used to obtain the Twitter users that form the "Random" cohort. Then, we discuss the possible influences of changes in natural light that could influence the circadian rhythm of individuals, such as the distribution of dawn, sunrise, sunset and dusk times over our cohorts and Daylight Savings Time (DST). Subsequently, we show that there is no phase-shift between the two cohorts that we study. Finally, we discuss the selection process for the tokens that we used in our content analysis and the subsequent results of this analysis.

1 Time of diagnosis interpretations

We queried the IUNI Observatory on Social Media (OSoMe) [4] for tweets that contained both variants of the terms '`depress`' and '`diagnos`'. Of this set of tweets, we retained only those that matched the regular expression: '`I.*diagnos.*depress`' since they were most likely to express a self-reported diagnosis such as "I was recently diagnosed with depression".

We then manually determined whether each such tweet actually contained a valid "diagnosis" statement, to remove jokes, quotes, and non-self-referential statements (e.g. "I just heard that Captain America was diagnosed with depression"). In total, we obtained 1466 tweets posted by 1211 individuals that stated a valid self-referential statement of a depression diagnosis.

Moreover, we also annotated whether the individuals provided an indication as to when they were diagnosed, e.g. "I was diagnosed with depression on October 4, 2018" or "I was diagnosed with depression *1 month ago*". 509 out of 1466 valid 'diagnosis tweets' contain such a Time of Diagnosis (TOD) indication. These TODs could be exact ("March 1st of last year", "today", etc.) or approximate ("3 weeks ago", "last Fall", "sometime last year", etc). To standardize our interpretations of the TOD, we defined a set of rules to translate the TOD (exact or approximate) to an interval of days in which the diagnosis most likely occurred based on (1) the date of the diagnosis tweet, (2) the TOD, and (3) the precision of the TOD ("days". "weeks", "months", "years"). Overall, the TOD interval was defined as (diagnosis tweet date - stated TOD)$\pm\frac{1}{2}$ units. For example, if an individual stated "2 weeks ago" in a tweet on 2018-02-22, this is interpreted as a diagnosis interval that spans from 2018-02-05 to 2018-02-11, i.e. the week that contains the 2018-02-08 (two weeks before the date of the tweet). Conversely, if a diagnosis tweet stated "I was diagnosed with depression on March 1st, 2019" the diagnosis interval was exactly 1 day, namely 2019-03-01.

See the Jupyter notebook called `time_conversion.ipynb` for the exact mappings of the diagnosis intervals.

1.1 Distribution of the self-reported times of diagnosis

Based on the aforementioned 509 TOD indications, we are able to obtain an interval in which the diagnosis occurred for 405 individuals. Given that we analyze only the individuals for which we have local time information, we will only analyze the 253 individuals for which we have time zone information. Note Twitter started operation in 2006 and due to a limitation of the Twitter API, the retrieved timeline of an individual can only contain a maximum of their 3,200 most recent tweets. These factors limit the time span of the timelines we retrieved. A diagnosis tweets can therefore refer to a time interval that falls outside the bounds of the individual's timeline, e.g. "I was diagnosed with depression 40 years ago". Hence, as a last step, we limit our set of individuals to those for which the self-reported diagnosis time occurred within their Twitter timeline. This gives us a total of 93 individuals.

For these 93 individuals, a total of 30 individuals have referenced an exact date of their self-reported diagnosis. A subset of 78 individuals (i.e., 83.87%) reported a diagnosis time that occurred within a year of the tweet that reported the diagnosis. In fact, the median and mean number of days between self-report and TOD for these users are 0 and 39.8 days respectively. For the other 63 individuals, the median and mean time between the self-report and the reported diagnosis is 183.5 and 353.49 day (about half a year and a year).

The most frequently occurring exact TOD is the date of the actual self-report, which is the case for 19 individuals. Examining the distribution of TOD intervals (not exact TOD dates), we find that the most frequently occurring expected TOD is 46 days before the diagnosis tweet (in particular 8 individuals reported a TOD of "recently").

These results support the conclusion that our inclusion criteria for the "Depressed" cohort selects individuals that either were likely depressed at the time of the self-report tweet or had recently been depressed ([7]).

Fig. S1 shows the distribution of the difference between the tweet containing the self-report and the TOD according to our mapping for these 93 individuals. The inset shows a close-up of the aforementioned 78 users who have an expected diagnosis interval size that is less than a year. The black lines and dots indicate the size of the actual diagnosis intervals.

2 Random Sample design

We construct a cohort of random Twitter users to use as a baseline for comparisons to the "Depressed" cohort. Since the platform might be used differently over time, we construct our random sample in such a way that the distribution of account creation dates in our sample matches those of the "Depressed" cohort.

First, we choose three weeks as the basis of our random sample design: September 1-8 2017, March 1-8 2018 and September 1-8 2018. For these weeks, we obtain a random sample of tweets from the IUNI Observatory on Social Media (OSoMe)[4]. The individuals who posted these tweets were taken as the starting set for the random sample, which gives us a set of 588,356 unique individuals.

From this starting set, we then select all individuals that have specified their location and that are not yet in our "Depressed" cohort, retaining 387,509 individuals. Next, we retain all individuals for whom we can translate their self-described location to a specific time zone, using the the same location-to-time zone mapping developed during the formation of the "Depressed" cohort. This operation results in a sample of 71,277 remaining individuals for whom we could determine a time zone.

We then sample these remaining individuals such that the distribution of their profile creation months matches that of the "Depressed" cohort (removing individuals with creation dates that do not occur in the Depressed cohort). Following this procedure, we retain a set of 9,525 individuals and retrieve their Twitter timelines.

Some of these individuals may have changed their profile settings to private or have deleted their account, so we were not able to collect timelines for the entire intended sample of individuals. In total, we collected the timelines of the 9,121 individuals and obtain 24,992,122 tweets for our random sample data set. These tweets range from 2007-08-24 20:36:05+00:00 to 2019-02-08 12:51:43+00:00.

After performing the same filtering steps on these tweets as we applied to the "Depressed" cohort, i.e. retaining only English tweets, removing retweets and tweets that mention either '`depress*`' or '`diagnos*`',

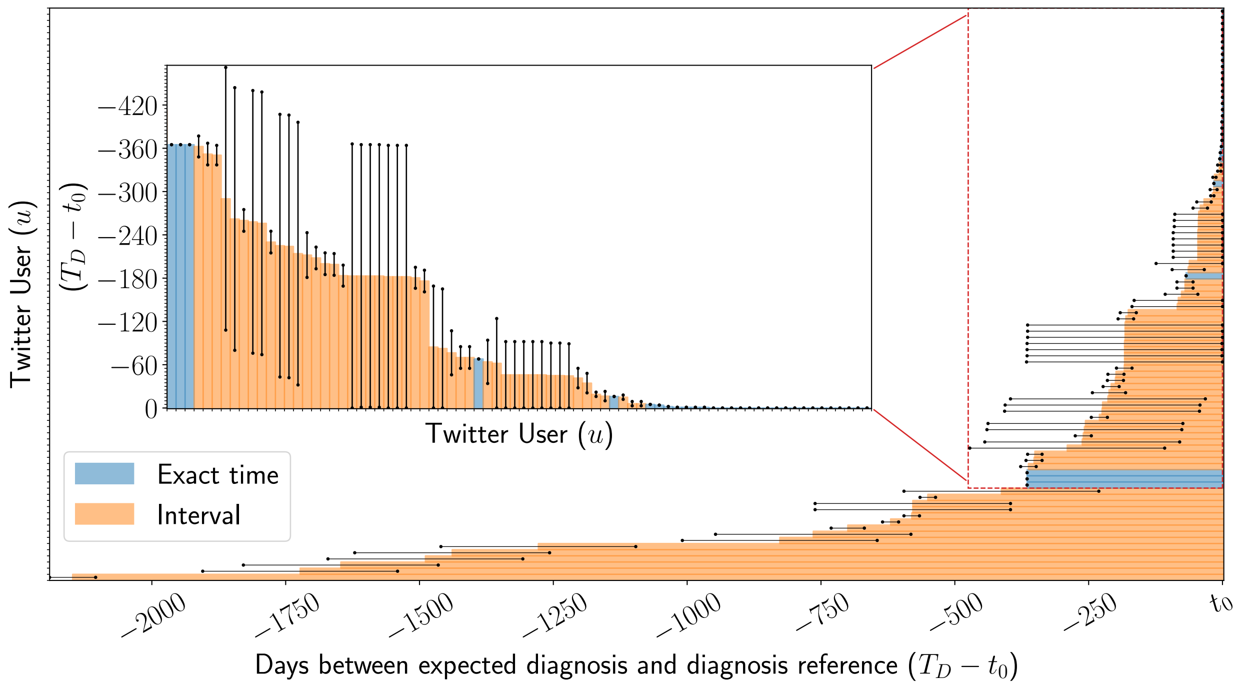

Figure S1: **Distribution of difference between the expected time of diagnosis and the diagnosis reference in the "Depressed" cohort.** The blue colored intervals indicate individuals that self-reported an exact date of diagnosis and the orange colored intervals indicate users for which we determined an interval in which their self-reported diagnosis occurred based on their time indication in their tweet. Furthermore, the black dots and lines indicate the exact distribution of the diagnosis window for each individual. The inset shows a close-up of all diagnosis intervals that are within a year from the self-report tweet.

we end up with a cohort of 8,498,574 tweets that are posted by 8,791 Twitter users. We will refer to these individuals and tweets as the "Random" cohort.

3 Natural changes affecting circadian rhythms

Since the individuals in our "Depressed" and "Random" cohort can live in different geographic areas with different daylight cycles, we examine the degree to which our results are influenced by local changes in daylight and their variation between the individuals in our cohorts.

3.1 Daylight cycles

To control for the effects of daylight cycles for each of the individuals in our cohorts, we retrieve the user-defined locations of each tweet to derive the time of dawn, sunrise, sunset and dusk at the location that the tweet was posted from using the python packages *Astral*[1] and *GeoPy*[6]. From here on, we refer to the times of dawn, sunrise, sunset and dusk as *sun times*.

We calculate sun times for each individual in our Depressed and Random sample, resulting in a distribution of sun times which we can characterize by its median, mean and confidence interval. However, these distributions can vary depending on the construction of our "Depressed" and "Random" samples. To determine the expected degree of variation of these results, we randomly re-sample the individuals in each cohort 10,000 times (with replacement) and recalculate the distribution characteristics for each re-sample. The resulting distribution yields a 95% confidence that reflect the uncertainty in our estimates resulting from random changes in the geographic location of our cohort samples.

The complete distribution of the sun times for both cohorts is displayed in Fig. S2. We annotated the median sun times for the "Depressed" cohort in Fig. 1.

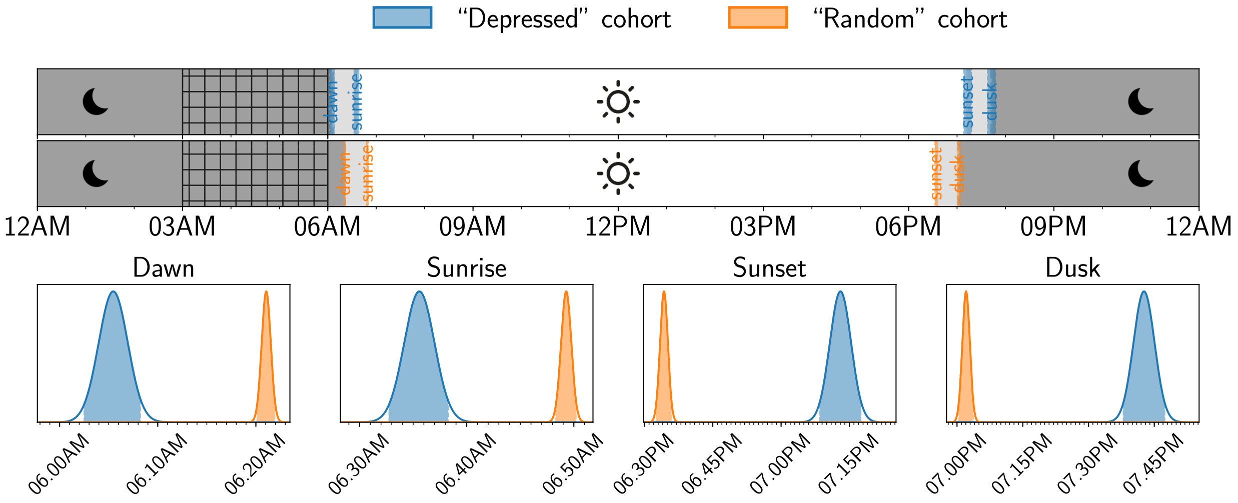

Figure S2: **Comparison of the bootstrapped sun times for both cohorts ($n = 688$ for the "Depressed" and $n = 8,791$ for the "Random" cohort).** The top rows indicate the 95% CIs for the sun times and the black checkered area indicates the times in the early morning when the activity patterns between both cohorts show significant differences. The gray shadings indicate the amount of daylight during these intervals. The bottom panels display the distribution and the 95% CI of the bootstrap of the sun times for both cohorts.

The top two panels in Fig. S2 display a colored band which indicates the 95% CI of each of the sun times for both cohorts. Although there are clear differences between the two cohorts in terms of the sun times, both do not align with the differences in activity levels in the early morning that are indicated by the black checkered overlay in Fig. S2. The panels in the bottom row show the distribution of the bootstrapped sun times. The difference in width of these distributions can be explained by the number of individuals in each cohort. Moreover, although the distributions do not overlap, the actual times are less than 20 minutes apart for the morning hours and less than 45 apart for the evening hours. These differences are most likely the result of a difference in latitude of the locations for the individuals that are contained in both cohorts.

3.2 Daylight Savings Time

Our user timelines can span across multiple changes from Standard Time to Daylight Savings Time (DST) which may bias some of the observed changes in circadian fluctuations of activity levels. Therefore, we determine for all tweets in our cohorts whether they were posted when DST was in effect or not. To determine this, we use the time zone that we derived for each individual based on the location information that they provided on their Twitter profile.

For the "Depressed" cohort, 645,249 and 397,589 tweets were posted when DST was in effect and not in effect, respectively, and for the "Random" cohort, 3,700,802 and 4,797,772 tweets were posted when DST was in effect and not in effect, respectively. Therefore, it is logical that the CI of the sun times for all tweets in the cohort is shifted towards the DST CIs, as is depicted in Fig. S3 and one would expect that the CI of the sun times for all tweets in the cohort is shifted towards the 95% CIs of the cycles where DST not in effect, as is depicted in Fig. S4.

However, when we calculate the activity levels throughout the day using only tweets that were posted when DST was in effect or not in effect, we obtain no differences in the activity levels throughout the day, as is shown in Figs. S5 and S6. This shows that the activity cycle of the individuals in both cohorts is not influenced by changes in the daylight cycle. In other words, we observe no effect on circadian activity

patterns from changes to DST.

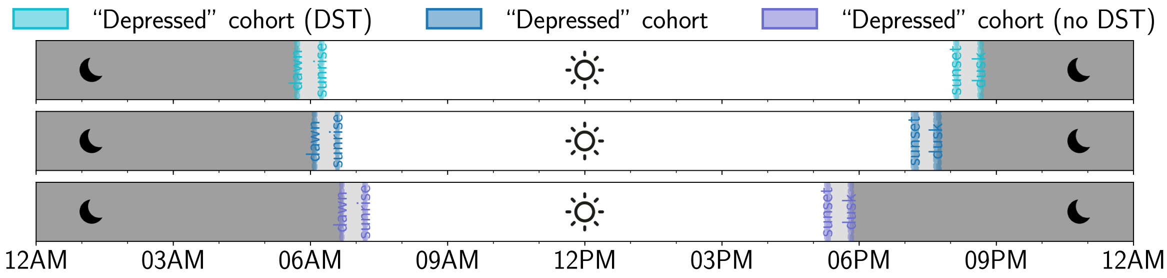

Figure S3: **Influence of Daylight Savings Time on the sun time distribution of the "Depression" cohort.** The top row shows the 95% CI of the sun times using only tweets that were placed when DST is in effect, the middle row shows the 95% CI of the sun times using the entire cohort and the bottom row shows the 95% CI of the sun times using only tweets that were placed when DST is not in effect.

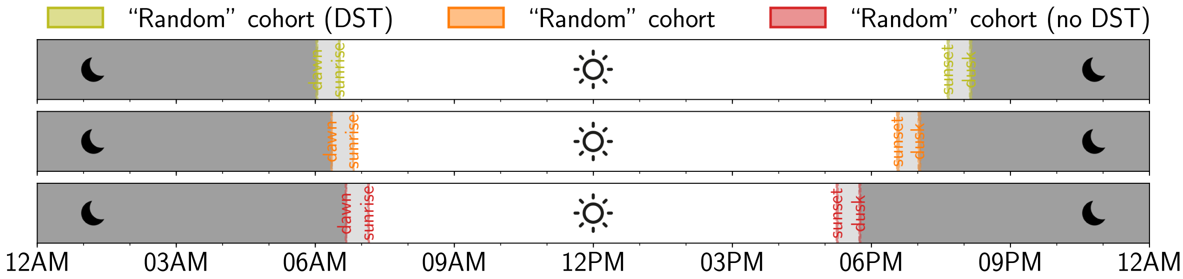

Figure S4: **Influence of Daylight Savings Time on the sun time distribution of the "Random" cohort.** The top row shows the 95% CI of the sun times using only tweets that were placed when DST is in effect, the middle row shows the 95% CI of the sun times using the entire cohort and the bottom row shows the 95% CI of the sun times using only tweets that were placed when DST is not in effect.

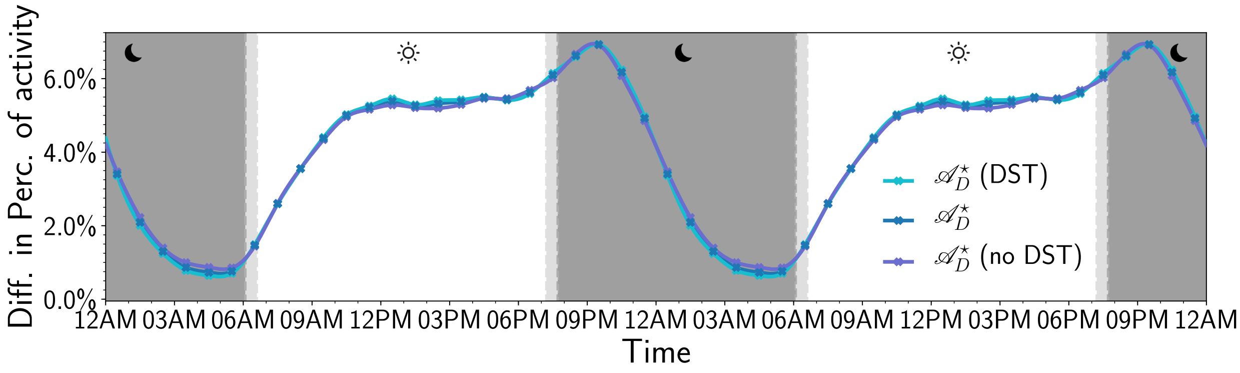

Figure S5: **Influence of DST on the activity levels of the "Depression" cohort.** Activity levels for the "Depressed" cohort using tweets posted when DST was in effect (in light blue), all tweets (in blue), and tweets posted when DST was not in effect (in purple), respectively. The gray annotations show the median of the bootstrapped sun times for the "Depressed" cohort.

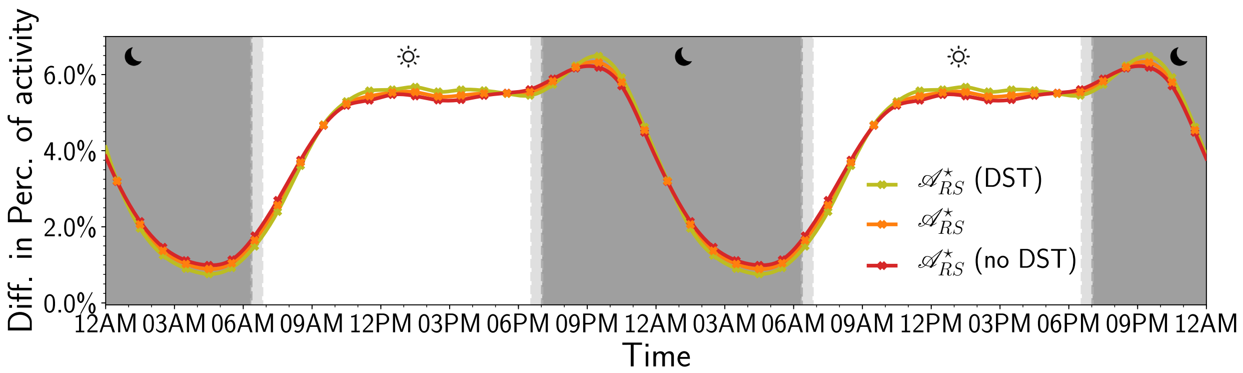

Figure S6: **Analyzing the influence of DST on the activity levels of the "Random" cohort.** Activity levels for the "Random" cohort using tweets posted when DST was in effect (in yellow), all tweets (in orange), and tweets posted when DST was not in effect (in red), respectively. The gray annotations show the median of the bootstrapped sun times for the "Random" cohort.

4 Testing for a phase-shift between cohorts

In the comparison of the activity levels of the "Depressed" and "Random" cohorts, we also check whether these time series display a phase-shift. We do this by calculating the Pearson correlation coefficient between the "Depressed" time series and a shifted version of the "Random" time series for time shifts between -12 and 12 hours. The top panel of Fig. S7 displays the obtained correlation scores, of which the maximum value is 0.9929 in which the time series of the "Random" cohort is shifted by zero hours. The significance threshold for the comparison that we perform is 0.496, given the fact that we use 24 data points. From Fig. S7, we see that all shifts between -3 and 3 hours have significant levels of correlation between the two time series. Therefore, we display the corresponding time series with the -3, 0, and 3 hour shifts in the bottom panel of Fig. S7 in green, orange, and red respectively. These results imply that there is no phase-shift between the time series of our two cohorts.

5 Content analysis

We extract content indicators from our tweet as follows. First, the tweets are tokenized (split into separate tokens) using the built-in `TweetTokenizer` of the *NLTK*[2] package. Next, all tokens that start with a capital letter and have no further capitals are converted to a non-capitalized form, with the exception of "I". Finally, any contractions that have a unique meaning, e.g. "I'm" or "you'll", are converted to their non-contracted counterparts, e.g. "I am" or "you will". Ambiguous contractions such as "he's", which can mean both "he is" and "he has", are not converted.

5.1 Selecting tokens for the content analysis.

To focus our analysis on tokens that are consistently used throughout the day (allowing for comparisons across different times), we determine the 250 most used tokens for each separate hour. Of these tokens, 187 tokens occur in the top 250 tokens for *every hour* throughout the entire day. This set of highly frequent and consistently used tokens is displayed in Table S1.

Two of the authors, who are clinical experts in cognitive-behavioral therapy (CBT), defined six topic categories for a sub-set of these tokens (see Table 2) that were deemed most relevant with respect to the cognitive factors affecting depression, namely "Personal Pronouns", "Rumination", "Negative Affect", "Questioning", and "Rigid Thinking". A sub-set of "Positive Affect'-tokens was defined as a control. These categories align with previous work that analyzes the social media content of depressed individuals [3, 5]. Next, some tokens were added to complement and balance some of the categories based on the expertise of the clinical expertise of the CBT experts. These added tokens are marked with an asterisk (*) in Table 2. Finally, since online

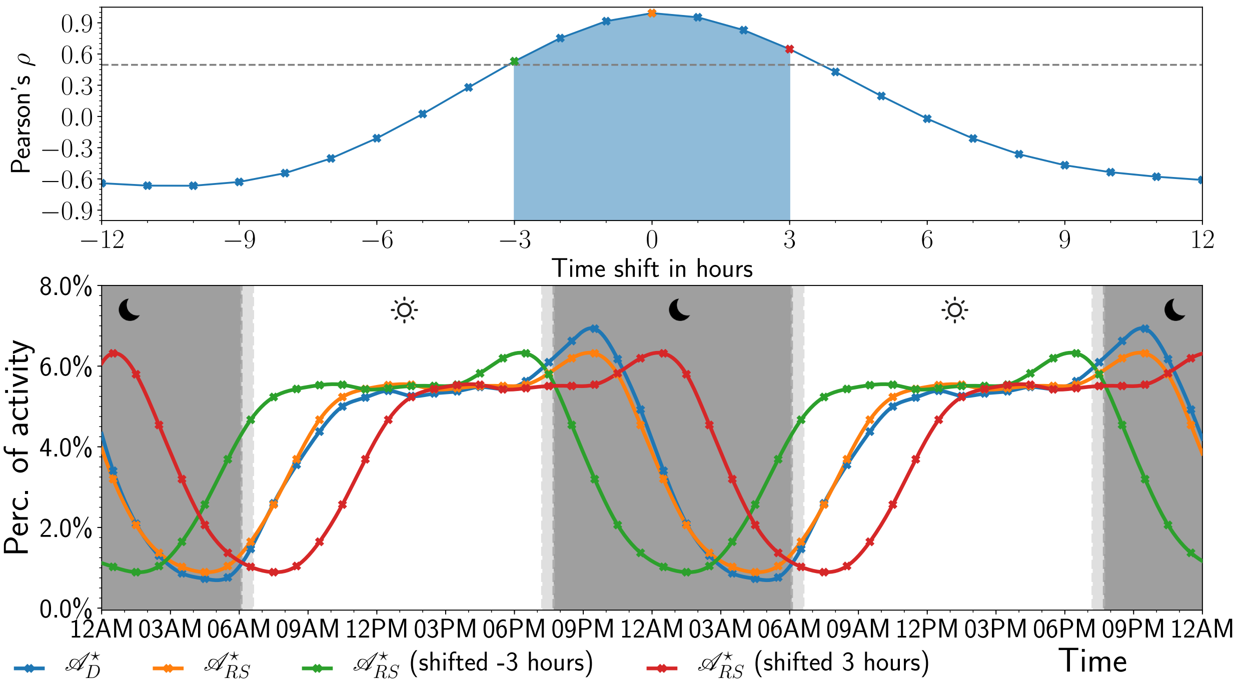

Figure S7: **Phase-shift test between the "Depressed" and "Random" cohorts.** The top panel shows the Pearson correlation coefficients between the time series of the "Depressed" cohort and a shifted version of the "Random" cohort. The gray dashed line indicates the significance threshold for the correlation coefficients and the colored area shows the shifts for which the correlation is significant. The green, orange, and red markers correspond to the time series that are shown in the bottom panel. The blue line in the bottom panels shows the time series of the "Depressed" cohort.

communication frequently involves the use of emojis, we added a set of emojis taken from the classification of hotemoji[1] to the positive and negative affect categories. We only included emojis with obvious positive and negative valence which are also indicated by an asterisk (*) in Table 2. This procedure resulted in a total of 76 tokens distributed over six categories.

5.2 Comparing activity level differences with token prevalence

We assess whether the observed differences in activity patterns between the "Depressed" and "Random" cohorts correspond to changes in content across the mentioned token categories. For example, are tokens indicating Rumination more prevalent when the Depressed cohort is significantly more or less active? We define the prevalence of a token t in a cohort G (i.e., D for "Depressed" or RS for "Random") as the average frequency with which the token appears in the tweets $\mathcal{T}$ of the individuals in a cohort, i.e.,

$$f_G(t) = \frac{\sum_{u \in G} \sum_{\mathcal{T} \in T_u} \sum_{u \in \mathcal{T}_t} \mathbb{1}\,(u = t)}{\sum_{u \in G} |T_u|}, \tag{1}$$

where T_u denotes the timeline of individual u and $\mathcal{T}_t$ denotes the set of tokens that a specific tweet in T_u contains.

We further define $\mathcal{C}_x$ to denote all tokens within a given category, e.g. $\mathcal{C}_{PP}$ denotes the set of tokens in the Personal Pronouns category. Using these definitions, we can define the average prevalence for a category

[1]See `https://hotemoji.com/emoji-meanings.html`

Table S1: **Overview of tokens that are in the top 250 most used tokens for each our of the day.**

!	"	&	’	(	)	*	,	-	.
..	...	/	2	3	:	?	I	a	about
after	again	all	also	always	am	an	and	any	are
as	at	back	be	because	been	before	being	best	better
but	by	can	come	could	day	did	do	does	down
even	ever	every	feel	first	for	from	get	getting	go
going	good	got	great	had	happy	has	have	he	he’s
help	her	here	him	his	how	i	if	in	into
is	it	it’s	just	keep	know	last	let	life	like
lol	look	love	made	make	man	many	me	more	most
much	my	need	never	new	no	not	now	of	off
oh	on	one	only	or	other	our	out	over	people
please	really	right	said	same	say	see	she	shit	should
so	some	someone	something	still	take	than	thank	thanks	that
that’s	the	their	them	then	there	these	they	thing	things
think	this	those	time	to	today	too	u	up	us
very	want	was	way	we	well	were	what	when	where
who	why	will	with	work	would	year	years	you	your
“	”	. . .	❤️		😂	😭			

as $\frac{1}{|\mathcal{C}_x|} \cdot \sum_{t \in \mathcal{C}_x} f_G(t)$ and the token prevalence ratio of a category as

$$PR(\mathcal{C}_x) = \frac{1}{|\mathcal{C}_x|} \cdot \sum_{t \in \mathcal{C}_x} \frac{f_D(t)}{f_{RS}(t)}. \tag{2}$$

Table S2 shows the distribution of token prevalence for all categories and both cohorts as well as the relative difference between the two cohorts. The last column of Table S2 clearly shows that on average, the tokens in each category are used more frequently by the “Depressed” cohort than the “Random” cohort, since all values in the right column of Table S2 are larger than 1. These findings are in line with machine learning approaches where certain word-use has been shown to successfully predict whether Facebook users suffer from depression [3, 5].

Table S2: **Distribution of token prevalence for all considered categories across both cohorts and their relative difference.** Per category, we display the mean and the distribution of all values within this categories, indicated within brackets. The relative difference is calculated as the average relative difference of all tokens in the category.

Category $\mathcal{C}_x$	Token prevalence $\frac{1}{|\mathcal{C}_x|}\sum_{t\in\mathcal{C}_x} f_G(t)$, $[\min_{t\in\mathcal{C}_x}(f_G(t)), \max_{t\in\mathcal{C}_x}(f_G(t))]$ “Depressed” cohort	“Random” cohort	Relative difference $PR(\mathcal{C}_x)$
All Tokens ($\mathcal{C}_{ALL}$)	0.0313, [0.0002, 0.5519]	0.0219, [0.0001, 0.3064]	1.4885
Personal Pronouns ($\mathcal{C}_{PP}$)	0.1481, [0.0036, 0.5519]	0.0841, [0.0020, 0.3064]	1.7601
Positive Affect ($\mathcal{C}_{PA}$)	0.0189, [0.0014, 0.0669]	0.0142, [0.0007, 0.0501]	1.4593
Negative Affect ($\mathcal{C}_{NA}$)	0.0031, [0.0002, 0.0180]	0.0022, [0.0001, 0.0138]	1.6982
Rumination ($\mathcal{C}_R$)	0.0333, [0.0180, 0.0628]	0.0269, [0.0143, 0.0556]	1.2455
Questioning ($\mathcal{C}_Q$)	0.0345, [0.0130, 0.0563]	0.0284, [0.0107, 0.0506]	1.2319
Rigid Thinking ($\mathcal{C}_{RT}$)	0.0420, [0.0134, 0.1999]	0.0323, [0.0097, 0.1507]	1.3467

To further analyze this difference in word-usage between the cohorts, we examine the prevalence ratio on an hourly scale (denoted by $f_G^h(t)$ in which h denotes the hour of the day). Thus, we can express the hourly

token prevalence ratio between the two cohorts as

$$PR^h\left(t\right) = \frac{f_D^h(t)}{f_{RS}^h(t)}, \tag{3}$$

and the hourly token prevalence ratio per category as

$$PR^h\left(\mathcal{C}_x\right) = \frac{1}{|\mathcal{C}_x|} \cdot \sum_{t \in \mathcal{C}_x} \frac{f_D^h(t)}{f_{RS}^h(t)}. \tag{4}$$

Fig. S8 displays the values of this hourly token prevalence ratio for all tokens. The periods of time when the activity levels of the "Depressed" and "Random" cohort diverge are indicated by gray shaded areas. As shown, the hourly prevalence ratios per category follow a similar pattern. All tokens that we used in our selection are displayed in panel A of Fig. S8. The majority of the markers are above the gray line which indicates the point where prevalence ratios equal 1. In fact, 94.13% of the hourly prevalence ratios are greater than 1, which indicates why these tokens were useful as feature sets to predict depression [3]. The data-points below the line $RC = 1$ are of interest because they indicate where a taken is more prevalent among the random sample at that time. 52.94% of these cases pertain to tokens that belong to the Positive Affect category, which differs from all other categories in this respect.

All categories $PR^h(\mathcal{C}_x)$ exhibit consistent patterns of hourly token prevalence ratios, with the exception of Positive Affect. The latter shows an increased relative prevalence from 3AM to 6AM. We see a drop in positive affect and an increase in Rigid Thinking and Questioning from midnight to 3AM. This is followed by an increase in the use of tokens associated with Personal Pronouns and Negative Affect from 4AM to 6AM. Token use across all categories peaks from 5AM to 6AM, the early morning hours, indicating higher levels of rumination and self-reflection among individuals in the "Depressed" cohort, matched by an increase in Positive Affect tokens.

We stress that these differences occur in conjunction with *lower* activity levels for the "Depressed" cohort from 3AM to 6AM as indicated in Fig. 2. Even though depressed individuals are less active in that period, those that are active exhibit higher token use across all categories. Since our tokens were designed to indicate self-reflection and rumination (with the exception of "Positive Affect"), this pattern is indicative that wakefulness at that time is associated with negative psychological states.

References

[1] Astral. Read the docs: Astral. `https://astral.readthedocs.io/en/latest/`, 2019. Retrieved on August 27th 2019.

[2] Steven Bird, Ewan Klein, and Edward Loper. *Natural Language Processing with Python*. O'Reilly Media, Inc., 2009. ISBN 0596516495, 9780596516499.

[3] M. De Choudhury, M. Gamon, S. Counts, and E. Horvitz. Predicting Depression via Social Media. In *Proceedings of the 7th International AAAI Conference on Weblogs and Social Media*, ICWSM, pages 128–137. AAAI, 2013.

[4] Clayton A Davis, Giovanni Luca Ciampaglia, Luca Maria Aiello, Keychul Chung, Michael D Conover, Emilio Ferrara, Alessandro Flammini, Geoffrey C Fox, Xiaoming Gao, Bruno Gonçalves, et al. OSoMe: the IUNI observatory on social media. *PeerJ Computer Science*, 2:e87, 2016.

[5] Johannes C. Eichstaedt, Robert J. Smith, Raina M. Merchant, Lyle H. Ungar, Patrick Crutchley, Daniel Preoţiuc-Pietro, David A. Asch, and H. Andrew Schwartz. Facebook language predicts depression in medical records. *Proceedings of the National Academy of Sciences*, 115(44):11203–11208, 2018. ISSN 0027-8424. doi: 10.1073/pnas.1802331115.

[6] GeoPy. Read the docs: GeoPy. `https://geopy.readthedocs.io/en/stable/`, 2019. Retrieved on August 27th 2019.

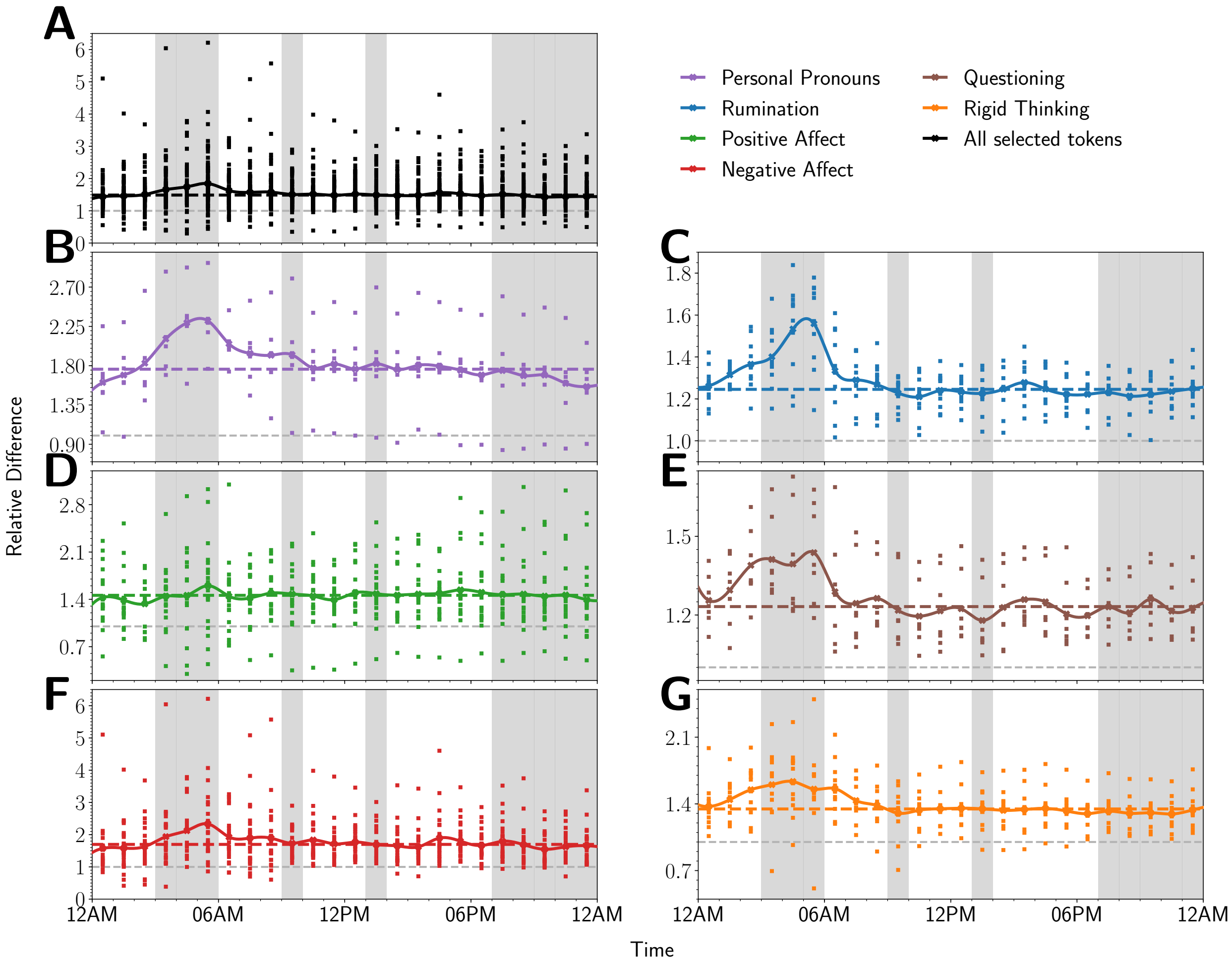

Figure S8: **Relative difference in token usage between the "Depressed" and "Random" cohorts.** The hourly prevalence ratios for all selected tokens ($PR^h(t)$) are depicted as dots. The solid lines display the cubic spline fit of the hourly average values for each category ($PR^h(\mathcal{C}_x)$) and the values are depicted as crosses. The colored dashed lines indicate the mean relative difference in token usage over all tweets for each category ($PR(\mathcal{C}_x)$) and the gray dashed lines indicate where the prevalence ratio equals 1. Furthermore, the gray shaded areas indicate the hours in which there is a significant difference in activity.

[7] J. Spijker, R. de Graaf, R.V. Bijl, A. T. Beekman AT, J. Ormel, and W. A. Nolen. Duration of major depressive episodes in the general population: results from The Netherlands Mental Health Survey and Incidence Study (NEMESIS). *The British Journal of Psychiatry*, 181:208–213, 2002.
